# *Notes from the Field:* Readiness for Use of Type 2 Novel Oral Poliovirus Vaccine in Response to a Type 2 Circulating Vaccine-Derived Poliovirus Outbreak — Tajikistan, 2020–2021

**DOI:** 10.15585/mmwr.mm7109a4

**Published:** 2022-03-04

**Authors:** Patrick O’Connor, Shahin Huseynov, Carrie F. Nielsen, Faizali Saidzoda, Eugene Saxentoff, Umeda Sadykova, Patricia Kormoss

**Affiliations:** ^1^World Health Organization Regional Office for Europe, Copenhagen, Denmark; ^2^Global Immunization Division, Center for Global Health, CDC; ^3^Ministry of Health and Social Protection of the Population of the Republic of Tajikistan, Dushanbe, Tajikistan; ^4^World Health Organization Country Office for the Republic of Tajikistan, Dushanbe, Tajikistan.

On January 13, 2021, a vaccine-derived poliovirus type 2 (VDPV2) was identified by the Regional Reference Laboratory for Polio in Moscow, Russia* in a specimen from a patient with acute flaccid paralysis (AFP) in Jaloliddin Balkhi district, Khatlon Region, in Tajikistan. Paralysis onset occurred on November 22, 2020. On February 6, 2021, a second, genetically linked VDPV2 paralytic case, with onset of paralysis on January 17, 2021, was confirmed from Khatlon Region in the neighboring Vakhsh district, indicating local transmission. Genetic sequencing of the isolate by the Regional Reference Laboratory for Polio in Moscow found a 20-nucleotide divergence from Sabin vaccine virus strain, and a 14-nucleotide divergence from a circulating VDPV2 (cVDPV2) reported from Khikorgangi, Pakistan on December 7, 2020, which suggests undetected circulation for approximately 12 months (*1*). On the basis of high-quality AFP surveillance in Tajikistan, the researchers concluded these cases likely represent recent importation (*2*). During 2014, the Director-General of the World Health Organization (WHO) declared polio a Public Health Emergency of International Concern under the International Health Regulations; the isolation of any poliovirus requires immediate reporting and prompt response (*3*).

Children born after the global cessation of use of type 2–containing oral poliovirus vaccine (OPV) from routine immunization schedules in April 2016 have no mucosal immunity against type 2 polioviruses. Therefore, cVDPV2 outbreak immunization responses require the use of type 2–containing OPVs; however, in low-coverage settings, use of type 2 oral poliovirus vaccine increases the risk for seeding^†^ of new cVDPV2 emergences (*1*,*4*). Current type 2–containing poliovirus vaccines are Sabin strain monovalent type 2 oral poliovirus vaccine (mOPV2) and trivalent oral poliovirus vaccine (tOPV); tOPV is preferred where cocirculation of wild poliovirus 1 and cVDPV2 occurs. To mitigate new seeding events, WHO granted Emergency Use Listing status for a recently developed, genetically stabilized, novel OPV type 2 (nOPV2) during November 2020. The Tajik Ministry of Health and Social Protection of the Population (MoHSPP), in consultation with partners, conducted a rigorous risk assessment and determined that nOPV2 was the best vaccine outbreak response option that also served to protect the polio-free status of the WHO European Region. MoHSPP completed and documented the 25 Emergency Use Listing readiness criteria for the initial use phase^§^ for vaccine release in 8 weeks, which was then authorized by the WHO Director-General, making Tajikistan the first country outside the WHO African Region to use nOPV2 (*5*). MoHSPP incorporated nOPV2 into three rounds of outbreak response, including supplementary immunization activities (SIAs) (Figure). The targeted age group for rounds 1 and 2 was children aged 0–65 months and for round 3 was children aged 0–55 months.

**FIGURE:**
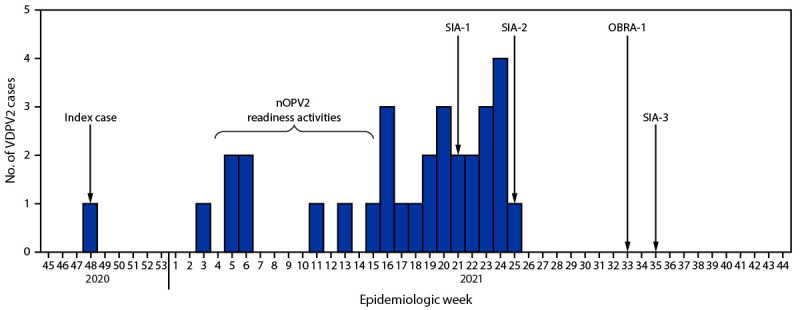
Circulating vaccine-derived poliovirus type 2 cases, novel oral poliovirus vaccine type 2 readiness activities, and outbreak supplementary immunization activities — Tajikistan, 2020–2021*^,†^ **Abbreviations**: nOPV2 = novel oral poliovirus vaccine type 2; OBRA-1 = first outbreak response assessment; SIA = supplementary immunization activity; SIA-1 = first SIA; SIA-2 = second SIA; SIA-3 = third SIA; VDPV2 = vaccine-derived poliovirus type 2. * Date of onset of paralysis for the index case: November 22, 2020; nOPV2 readiness activities: February 10–April 11, 2021; first nOPV2 SIA: May 31–June 6, 2021; second nOPV2 SIA: June 29–July 3, 2021; third nOPV2 SIA: August 30–September 4, 2021; OBRA-1: August 16–20, 2021. ^†^ National Expanded Program on Immunization data from weekly acute flaccid paralysis surveillance, Tajikistan, 2020–2021.

A total of 31 cVDPV2 cases were confirmed during November 22, 2020–June 26, 2021, with none occurring after the second SIA; virus was also isolated from close contacts of AFP cases, community-based stool collection surveys, and environmental samples.^¶^ The geographic spread of cVDPV2 included 10 districts within Khatlon Region, and in a broad central belt including Dushanbe, the capital. The first Outbreak Response Assessment was conducted during August 16–20, 2021, and an additional nOPV2 SIA was recommended at the end of August 2021 to ensure that transmission had been interrupted. Despite the challenges related to responding to a cVDPV2 outbreak during the COVID-19 pandemic, MoHSPP imported and distributed nOPV2, trained staff members, and conducted high-quality outbreak response activities (assessed via lot quality assurance sampling**). These efforts by MoHSPP resulted in administrative coverage of >99%, following mop-ups, in all three rounds, in this first use of nOPV2 outside the WHO Africa Region.

## Footnotes

* Tajikistan does not have a national polio laboratory; therefore, specimens are transported to the Regional Reference Laboratory for Polio in Moscow, Russia for testing on a regular basis. During the COVID-19 pandemic, regularly scheduled flights to and from Tajikistan were interrupted, and this affected the transportation of specimens.

† Oral poliovirus vaccines are live attenuated virus vaccines and provide intestinal immunity; poliovirus replicates in the intestinal tract. The vaccine virus is excreted in stool and can spread from person to person. However, in communities with low immunization coverage, vaccine virus can circulate during an extended period leading to reversion to neurovirulence, which can result in paralysis identical to that caused by wild polioviruses. https://www.cdc.gov/vaccines/vpd/polio/hcp/vaccine-derived-poliovirus-faq.html

§ The 25 nOPV2 readiness criteria are in nine categories: 1) coordination; 2) nOPV2 approvals; 3) cold chain logistics and vaccine management; 4) AFP surveillance; 5) environmental surveillance; 6) safety monitoring; 7) advocacy, communication, and social mobilization; 8) laboratory; and 9) campaign operations. https://polioeradication.org/wp-content/uploads/2020/12/nOPV2-Readiness-Verification-and-Dose-Release-Process-20201208.pdf

¶ As part of the nOPV2 readiness criteria, an environmental (sewage) collection point was identified in Dushanbe. The first specimen was collected the week of February 7, 2021 (epidemiologic week 6). Testing of specimens was supported by the Regional Reference Laboratories for Polio in Islamabad, Pakistan and Bilthoven, Netherlands. Data are current as of August 13, 2021.

** Assessing vaccination coverage levels using clustered lot quality assurance sampling. https://polioeradication.org/wp-content/uploads/2016/09/Assessing-Vaccination-Coverage-Levels-Using-Clustered-LQAS_Apr2012_EN.pdf
